# Implementing behavior change in healthcare epidemiology and antimicrobial stewardship: The worst that can happen is you fail

**DOI:** 10.1017/ash.2023.206

**Published:** 2023-07-26

**Authors:** Gonzalo Bearman, Priya Nori

**Affiliations:** 1 Division of Infectious Diseases, Virginia Commonwealth University Health, Richmond, VA; 2 Division of Infectious Diseases, Department of Medicine, Montefiore Health System, Albert Einstein College of Medicine, Bronx, NY

## Introduction

*You can’t let fear paralyze you. The worse that can happen is you fail but guess what: You get up and try again. Feel that pain, get over it, get up, dust yourself off and keep it moving*. –**Queen Latifah**

In healthcare epidemiology and antimicrobial stewardship, the implementation of safety measures and the improvement of antimicrobial use require healthcare provider behavior change. No single best approach exists for changing practices. Strategies are multipronged and include varied levels of stakeholder engagement, multispecialty collaboration, education, assessment, feedback, accountability, behavioral nudges, and mandates. We explore important yet underemphasized themes for affecting change in healthcare epidemiology and antimicrobial stewardship with the goal of raising awareness and providing an additional path in the implementation journey.

## Evaluating what to change and why?

Evaluating specific aspects of human behavior requiring change and barriers and enablers to influencing these target behaviors are fundamental to successful change implementation.^1^ Trial and error or provider education alone is not sufficient to affect the changes we seek and cost valuable time and energy. Starting with the fundamental questions of “what” and “why” from implementation science framework can help guide higher impact interventions and policies in health care.^1,2^ Hospital epidemiologists and antimicrobial stewards may not possess the expertise but should collaborate with institutional or regional experts in implementation science and seek to understand its fundamental concepts and how these can be applied to daily practice.^2^

In the book *Start with Why?* by Simon Sinek, all leadership decisions should begin with the critical question Why?^3^ This defines the underlying principle to guide all decisions and consequent actions. For matters related to antimicrobial stewardship and healthcare epidemiology, the guiding principle is *primum non-nocere*, or avoiding harm. Additional motivating factors include an awareness of organizational safety priorities, existing financial, and information technology and personnel resource challenges. Clarity on potential opportunity costs of both action and inaction regarding high stakes decisions must be clearly stated. Moreover, these decisions must be philosophically sound with well-articulated and transparent expectations of resources, effort, timeline, accountability, and outcomes.

## Leveraging high-quality data is necessary yet not sufficient: employ the power of persuasion

It is said that the data should do the talking, but this is an oversimplification as data alone can fall short on persuasion.^4^ Moreover, if high quality are not available, such as during a public health emergency, leaders must make decisions with the best available evidence and protect themselves and their teams from moral injury that may occur if desired outcome of decisions is not achieved.^5^

Although healthcare infection prevention and stewardship data guide policy and interventions, without effective narratives and mission centered talking points which resonate equally with frontline personnel and hospital executives, even high-quality data may not move decision makers. Of note, persuasion can be a tool to garner genuine support for patient safety initiatives without being perceived as a manipulative tactic.

For maximal success, leaders must harness the art of persuasion, which is rooted in the psychology of relationships and influence.^4,6^ These are summarized as six principles, employed in varying combinations to maximize their impact^6^ The first is the Principle of Liking. People like those who like them and cultivate relationships based on similarities and shared experiences, common goals, and mutual respect. These factors serve to engage and establish a connection, making it easier to generate momentum for initiatives, which individuals are already inclined to support. The second is the Principle of Reciprocity, in which you model what you wish to receive. Under this framework, leaders are more likely to elicit desired behaviors from colleagues by displaying them first. Next is the Principle of Social Proof or maximally leveraging peer pressure and peer to peer comparisons to promote the desirable behaviors. The fourth is the Principle of Consistency, where people’s behaviors align with their commitment to their personal mission and purpose, which is inviolate. Organizational research suggests that written mission statements are more powerful when they are made public. When initiatives are directly linked to organizational mission statements, consistency of purpose and action prevails.^4,6^ The Principle of Authority stems from the importance of deference to experts and people’s desire to follow the lead of legitimate experts. Leaders should know when to defer to expertise outside of their content areas to harness persuasion effectively. In epidemiology and stewardship, especially in decisions involving patient outcomes, know the limits of your expertise and when to defer to the expertise of other groups. Last is the Principle of Scarcity, in which a persuasive individual highlights the unique benefits of a desired intervention, one that is beneficial to an individual and the group.

## Be clear on expectations, do not oversell outcomes

A major challenge in change management is setting clear expectations, maintaining humility, and not over selling outcomes. It is estimated that approximately 3% of all hospitalized patients will experience a healthcare-associated infection (HAI) yet achieving a state of no HAIs is a highly aspirational and unrealistic goal with the current infection prevention science and stringent HAI definitions.^7^ As previously published, the only way to eliminate HAIs is to not admit patients to the hospital.^8^ The extent to which HAIs are preventable remains debatable, but recent publications suggest that up to 75% are preventable when known risk reduction measures are reliably implemented.^9,10^ Therefore, be realistic and manage expectations when communicating the deliverables of your IP teams to hospital leadership to avoid conflicts later.

Similarly, while no cases of hospital-onset *C.difficile* infection (CDI) is an aspirational goal, administering no doses of antibiotics in hospitalized patients is not. Antibiotic use is a well-established risk factor for CDI, and the goal of stewardship programs is to reduce unnecessary use, not eliminate all use, otherwise the hospital would fall short of meeting other benchmarks for sepsis care, surgical site infection prevention, etc.^11^ As pertains to CDI, a realistic and specific deliverable of stewardship programs effectively communicated to hospital leadership is the following: “Our team will implement an electronic antibiotic time-out program to reduce excess days-of-therapy of broad-spectrum antibiotics on non-teaching services by 15% within 6 months.”

After starting with Why, a deliberate decision-making framework is required to manage expectations and anticipated outcomes. In the book *Decisive: How to Make Better Decisions in Life and Work*, by Chip and Dan Heath, the authors explore practical tools for better decisions in life and work.^12^ An early step in the decision process requires the question “rather than do this OR that, is there is a way to do this AND that?” Next, beware of narrow framing, confirmation bias and short-term emotion, which can result in bad decisions and *overconfidence* on how the future will unfold. All decisions must be reality tested with counterpoints, preferably by a diversity of stakeholders. Managing expectations thus requires communication so that all are clear on the decision process, the implementation steps, the associated timeline, the measurable goals, and the anticipated outcomes. Failure to match expectations with reality almost certainly results in an erosion of trust and confidence.

## Beware of ineffective meetings, be a content expert, and run meetings with skill

“If you had to identify, in 1 word, the reason why the human race has not achieved and never will achieve, its full potential, that word would be *meetings*” – Dave Barry, Humor Columnist

In health care, task forces, and other meetings are frequently assembled to address safety issues, such as elevated HAI rates; however, these groups can become bloated, may be run by administrators instead of content experts, and may “reinvent the wheel” by redefining well-known HAI risk factors and reduction strategies. These meetings are ineffective without clearly defined implementation plans, deliverables, timelines, and accountability. As a result, implementation and progress are hindered.

Excessive workplace meetings, where creativity and productivity commonly “go to die,” are explored in the book *The Surprising Science of Meetings,* by Steven Rogelberg.^13^ Although evidence suggests that the number of meetings is increasing and that poorly run meetings are abundant, eliminating all meetings is not a viable alternative. Effective meetings respect time (frequency, start, and end times), include the appropriate type and number of individuals with a diversity of backgrounds, perspectives, and training, have an agenda with clearly defined goals, allow appropriate interpersonal dynamics (skilled moderator, participant engagement, and sharing of perspectives), result in clear and understandable decisions, next steps, and timelines with accountability and a brief, written summary of the discussion (minutes).^13^ Healthcare epidemiologists, infection preventionists, and antimicrobial stewards must serve as content experts as well as effective and skilled meeting moderators and facilitators to maximize clarity of purpose, scope, and action.

## Beware of resistors and constipators: prepare to push beyond barriers

Within the same organization, competing interests and shifting priorities are a major challenge to behavioral change and safety implementation. Saint et al explored the impact of active resistors and organizational constipators on HAI efforts.^14^ Employing a qualitative survey methodology across 14 different healthcare systems, the researchers identified a pervasiveness of “active resistors,” personnel who open and vigorously oppose changes in infection prevention, along with organizational “constipators.” The latter are mid- to high-level executives who act as insidious barriers to change mid- to high-level executives who prevented or delayed certain actions without active resistance, thereby acting as insidious barriers to change by increasing the work required and delaying the implementation of evidence-based practices.^14–16^ Organizational constipators included chief medical and nursing officers who were indecisive and failed to follow-up on varying aspects of initiatives resulting in the loss of project energy and momentum.

In a mathematical model assessing the financial impact of resistors and constipators in an academic medical center, every 6-month delay in improvement of CHG bathing compliance resulted in approximately 11 preventable CAUTIs and an additional cost of $11,000.^15^ Every 6-month delay in implementing standardized central-line bundle kits resulted in approximately 10 CLABSIs and an additional $715,000 in costs.^15^ Overall, during the 5-year period, 102 CLABSIs and 105 CAUTIs were potentially preventable, with a savings of approximately $7.2 million through CLABSI prevention and $115,000 through CAUTI prevention .^15^ Everyday constipators we encounter in our implementation journeys may include mid-level administrators without patient care backgrounds who refuse to fund growth initiatives with potential to improve patient safety but do not increase hospital revenue, such as antimicrobial stewardship and infection prevention programs. In academic medicine, respected and long-standing departmental leaders who refuse to adopt or support new innovations, or actively resist efforts of junior faculty to challenge the status quo due to perceived inconvenience or threat to leaders’ influence maybe be perceived as constipators.

Collectively, resistors and constipators either decelerate the pace of change or effectively block efforts in patient safety. Unfortunately, no single best strategy exists for overcoming resistors and constipators. Commonly, multilevel interventions are required to push beyond barriers posed by organizational resistors and constipators. Potential successful approaches include educational initiatives, audit and feedback, marketing, use of media, the employment of champions, facilitators, and opinion leaders.^16^ Obtaining buy-in early in the dissemination process or transitioning them to other roles if buy-in cannot be achieved.^16^ As part of the change implementation journey, 1 must be nimble, and both mentally and strategically prepared for the inevitable barriers posed by active resistors and organizational constipators.

Cognitive biases may also create resistance to behavioral change in stewardship and infection prevention. Cognitive biases can be managed with more mindful decision making (eg, checklists), improving one’s one biases, and designing an environment which facilitates more accurate decision making (eg, clinical decision support tools). Langford et al. state that a basic understanding of cognitive biases can help explain why certain stewardship interventions are more effective than others and may inspire more creative strategies to ensure optimal patient care.^17^

## Seek positive deviants: leverage them as agents of change

According to the Positive Deviance Collaboration, “Positive Deviance (PD) is based on the observation that in every community there are certain individuals or groups whose uncommon behaviors and strategies enable them to find better solutions to problems than their peers, while having access to the same resources and facing similar or worse challenges”.^18^ Under this framework, when enabled by leadership, the implementation of change starts at the ground level of a community then is disseminated upward across an organization.^18^ After successful implementation of a given initiative, positive deviants engage and recruit others through deliberate meetings and discussions in a just culture (eg, a system of shared accountability by organizations which respond to employee behaviors in a fair and just manner) with leadership oversight.^18–21^ From this emerges a collaborative network for enhanced dissemination and implementation.^19^

A growing body of literature in infection prevention and control suggests that positive deviance is an effective strategy for various implementation initiatives. Alzunitan et al published a systematic review of positive deviance in infection prevention and control.^22^ In total, 14 observational, quasi-experimental studies were included in the final analysis. Eight studies reported improvements with hand hygiene and positive deviance strategies. For example, a multicenter positive deviance initiative resulted in overall hand hygiene compliance improvement from 47% in the preintervention phase to 62% in the positive deviance phase (*P* < .001).^23^ In addition, the incidence of device-associated infections per 1,000 patient-days and the median of length of stay between the pre and post intervention phase were significantly impacted (13.2 vs 7.5 per 1,000 patient-days, respectively, *P* < .039; and 11.0 vs 6.8 days, respectively, *P* < .001, respectively)^23^ As summarized in the meta-analysis, the overall HAI rates were measured in 5 studies, and positive deviance strategies for risk reduction were associated with improved HAI rates in 4 studies with positive deviance containing bundles successful in all studies.^22^ This systematic review suggests that positive deviance may be an effective strategy for improvement of both hand hygiene and HAI rates.

Positive deviance is a promising social empowerment tool for behavior change, which results in improved infection prevention. A classic example in healthcare is the frontline nurse and aspirational patient advocate who is not afraid to respectfully confront the senior physician entering and exiting a patient’s room without performing hand hygiene, or openly questions physicians’ antibiotic orders for asymptomatic bacteriuria in her patients without urinary symptoms.

Although future, high-quality studies are required to best define positive deviance process, epidemiologists and antibiotic stewards should seek positive deviants, or individuals and groups with a history of driving solutions-oriented behavior change within an institution. These are models of best practice which can be leveraged, with the support of senior leadership, to initiate and institutionalized change across an organization.

## Commitments from institutional leaders is key but keeping them focused is a challenge

In large organizations, creative ideas only go so far without endorsement from leadership. Vokes et al argue that hospital leadership plays a critical and specific role in the change implementation process.^24^ A culture of safety stars at the top. Under this framework, hospital leadership must establish a sense of urgency, communicate the vision, empower action, and provide the necessary resources to institutionalize change.^24^

Further, healthcare systems that fail to relentlessly pursue the highest standards of patient safety betray the public trust.^25^ However, the ultimate supervision of infection prevention and antimicrobial stewardship programs rests with hospital executives. Infection prevention and antimicrobial stewardship programs are neither staffed nor empowered for broad, system-level implementation and decision making—these tasks inevitably fall on senior leadership.^25^ Across organizations, even highly efficient executives fail when focus on the bottom line comes at the expense of people and broader safety goals.^26^ Similarly, keeping healthcare executives consistently focused on our agenda is an ongoing struggle due to their competing interests, like revenue and rankings. Leaders in healthcare epidemiology and stewardship must mitigate this challenge by regularly engaging and communicating effectively with chief medical and safety officers, nursing leaders, and CEOs.

## Beware of team dynamics: disengaged teams cannot engage and inspire others

Burnout is an ongoing threat to health care worker wellness and sustainability. In infectious diseases, physician burnout approaches 45% as reported in a subspecialty survey.^27^ A recent survey of the SHEA Research Network concluded that nearly half of the participants were experiencing burnout.^28^ Potential unique drivers of burnout in infection prevention include a heavy service component that is generally underappreciated by physician peers and staff and is difficult to balance with academic and clinical responsibilities.^29^ This may drive feelings of decreased efficacy and lack of value to the health system. Moreover, loneliness, isolation, and a lack of social connection are having a profound impact on the physical and mental health of Americans.^30^ This loss of connectivity may be seen with increasing reliance on virtual meetings for convenience, even as pandemic restrictions have lifted. No simple solution exists for minimizing burnout in healthcare systems and multilevel strategies are summarized elsewhere.^31^ Much like in other disciplines, work hour limitations and institutional and individual programs focused on mindfulness may improve well-being in the healthcare epidemiology and stewardship workplace.^32^ While these measures are necessary, they are not sufficient to eliminate healthcare worker burnout or maximize team function. Team dynamics are critically important for staff engagement and group performance. In a 2012 study across the Google organization, titled Project Aristotle, the importance of team dynamics is highlighted. Across 180 global teams, the make-up of the team was less impactful than team behavioral norms (dynamics). Creating psychologically safe environments, defined as a shared expectation that team members will not reject or shame each other for sharing ideas, taking risks, or soliciting feedback, resulted in enhanced team bonding and effective day to day interaction, which was the main driver of high functioning teams.^33^

High functioning teams require leaders who find ways to bring teams together, encourage and promote honest and compassionate conversations about challenges, frictions, ideas, and everyday aggravations to address the needs of both the individual and the team. Likewise, leaders of healthcare teams must encourage dialogue, which minimizes friction and negativity while maximizing creativity, collaboration, and synergy.

Leaders should also recognize that diverse teams consisting of individuals of different ages, genders, ethnicities, educational levels, and social and cultural backgrounds are more creative, productive, and make better decisions. In the corporate sector, companies with a diverse workforce are 35% more likely to experience greater financial returns.^34^ Likewise, studies in the healthcare sector show that a diversity of teams improves both patient care quality and financial performance of organizations.^35^

From all the above comes engagement and inspiration. A healthcare epidemiology or antimicrobial stewardship team which is uninspired, with decreased physical presence and disengaged in virtual meetings, will be challenged to inspire others, and affect change in the patient safety environment.

## Focus on individual and team resilience: harness the power of small wins

The multiple steps required of large-scale projects can be dauting and may paralyze both the onset and maintenance of effective action. Although the best strategies to drive and sustain innovations within organizations is debatable, an important component is the *progress principle.* ^36^ Under this paradigm, the greatest booster of emotions, motivations, and perceptions at work is making progress in meaningful work. When there is perceived progress at work, employees are more intrinsically motivated, with greater interest, enjoyment, and engagement.

Naturally, personal, and professional life is not immune to turbulence. However, effective leaders focus on progress in terms that are reasonable and readily measurable. Major wins and achievements are great yet rare. A focus on *minor* milestones, especially those that are meaningful and linked to the core mission of the team and the organization, is more realistic. A successful leader must pay attention to a team’s everyday activities and progress. This requires deliberate identification of the 1–2 significant, yet small events that move a project forward, with recognition of both team member input and identification of potential barriers. Feedback is positive, with the leader serving as a collaborative resource to identify critical next steps, minimize setbacks, and promote ongoing progress.^36^

To truly engage infection preventionists and antimicrobial stewards, help them recognize their own progress. Regular assessments of incremental goals with acknowledgement of completed steps in multilevel initiatives can help build momentum, maximize success, and potentially mitigate the impacts of burnout and dissatisfaction in the workplace. For instance, while reducing penalties due to excess surgical site infections after hysterectomy is the loftier institutional goal, this may seem daunting to an infection prevention team, especially when attempting to address all contributing factors at once. Instead, focusing on smaller more tangible goals and associated wins is more effective. Increasing compliance with a few aspects of the SSI prevention bundle at a time, such as timing or dosing of antibiotic prophylaxis, or completing recommended skin preparation may in aggregate help reduce SSIs and associated penalties. As a leader, acknowledgement of these incremental wins and championing the successes of your team to senior leadership will have longer lasting impacts.

## You’ve effectively implemented change, now what? sell your work at the podium

*Your audience gives you everything you need. They tell you. There is no director who can direct you like an audience. –* **Fanny Brice**

Healthcare epidemiology and stewardship professionals possess requisite training and analytical skills in their respective areas of expertise to execute daily operations and improve patient outcomes. However, the perception of their expertise by hospital stakeholders, their ability to connect with and convince frontline staff of best practices can be greatly enhanced by communicating persuasively and effectively at the “podium,” which may include presentations at virtual meetings, local townhalls and grand rounds, in-person conferences, or lectures to peers or junior learners at one’s own institution. The most memorable speakers at scientific meetings achieve this status via selective use of high-quality data presented through effective delivery and persuasion, without overwhelming the audience with excess information. Audience members must be convinced that the presenter truly believes in the content. They wish to be informed but also entertained.

Principles of adult learning dictate that learners are most engaged by brief, digestible, and engaging content with tangible take-home messages.^37^ Moreover, the current information overload atmosphere has overwhelmed us and fundamentally changed the way we process and internalize information.^38^ In the authors’ opinion, use of personal anecdotes, humor, references to popular culture or sports, or insertion of recognizable images, while seemingly distracting from the task of delivering scientific content, serve to better engage the audience, build trust, likeability, and leave a lasting impression on learners with the goal of improved retention of information. Therefore, leaders in epidemiology and stewardship are well served by inserting performative elements, like humor and anecdotes, into their presentations, without seeming inauthentic or rehearsed. These should feel natural and true to one’s own experiences, interests, and beliefs. This may be outside the “comfort zone” for some but an important skill nonetheless to relate to the audience and achieve truly effective communication.

## Institutionalize change and publish what you’ve learned: every project has a story to tell

After successful implementation of an organizational change with measurable impacts on healthcare processes or patient outcomes, publishing your findings can maximize the impact of your project and increase visibility for all involved. Additional benefits of abstract or manuscript publication include cementing relationships with collaborators for future projects or pursuit of grant funding, gaining the respect and trust of hospital executives who may greenlight future initiatives, and establishing one’s external reputation as a content expert, leading to invitations to present at society meetings, serve on panels, or expert committees.

Momentum from a project well done should be harnessed immediately for abstract submission to a scientific meeting or publication to a medical journal, as enthusiasm for project completion may extinguish with time. Common hurdles to publication include lack of perceived time, inexperience, not knowing where to begin, fear of rejection by peer reviewers for lack of novelty or strength of findings, striving for perfection, and the overall daunting nature of manuscript preparation. However, even projects with negative results or lack of impactful findings may still have a story to tell and contribute to the knowledge base. Readers (especially those from low resource settings) may benefit from a description of the processes and lessons learned. Multiple rounds of rejection are expected, and reviewer feedback should be received objectively to help improve the manuscript, however harsh. If strong belief in the mission of the project and accountability among team members remains, the outcome will ultimately be positive. Likelihood of successful completion of an academic project can increase by (1) including both experienced collaborators and trainees or junior faculty eager to contribute, (2) “crowdsourcing” sections of the manuscript by individual team member expertise, (3) establishing clear deadlines, and (4) holding brief and focused meetings to move the project forward and meet deadlines. Finally, identifying a journal which is a good fit for the project is essential. In addition to established journals in healthcare epidemiology, infection prevention, and stewardship, journal search engines can provide additional options.^39^ From there, exploring journal websites or placing an inquiry to journal editors is generally welcome and encouraged.

Finally, active dissemination of published work via social media outlets is an effective method for maximizing readership, citations, downloads, and overall impact of the publication. Furthermore, visual abstracts, disseminated on social media and inserted into presentations, increase the likelihood that the target audience engages with the publication in the form of retweets, sharing within organizations and professional networks.^40,41^ Social media offers a unique opportunity for authors to directly connect, initiate, and perpetuate dialogue on topics pertaining to the publication.^40,41^ Well crafted visual abstracts and social media engagement may increase the likelihood of further dissemination through special sections on the journal website or the journal’s podcast.^40–42^

**Figure 1.**
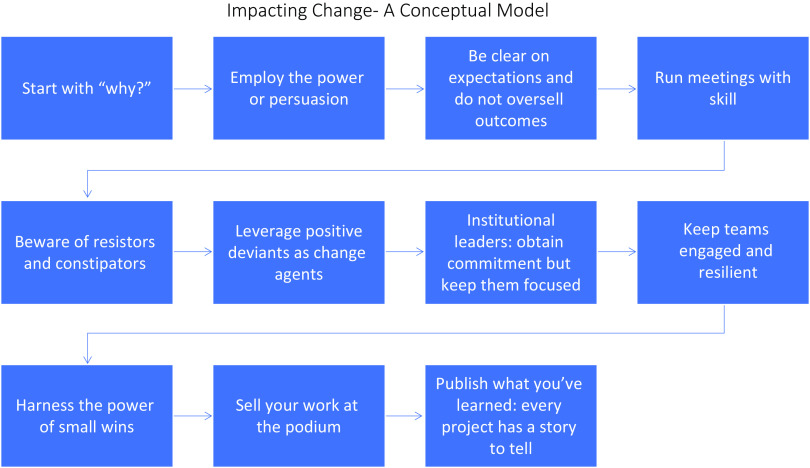
A conceptual model for impacting change in healthcare epidemiology and antimcrobial stewardship.

Putting it all altogether (Figure 1), for maximal persuasion and change implementation in antimicrobial stewardship and healthcare epidemiology, high-quality data are only the starting point. Healthcare epidemiology and antimicrobial stewardship leaders must strategically deploy the data by way of meaningful mutual relationships (Liking) across hospital leadership, pharmacy, medical, surgical, critical care colleagues, patients, and families. If a specific behavior is sought, change agents must always model the desired behavior (Reciprocity). Examples include being an antibiotic steward in prescribing practice and fully abiding by hand hygiene policies, wearing hand hygiene sensor badges, etc. Whenever feasible, leverage peer to peer comparisons (Social Proof) of antimicrobial prescribing and compliance with other reported safety measures. With all initiatives in patient safety, explicitly link the project to stated institutional goals (Consistency) and mission statements. Nearly all antimicrobial stewardship and healthcare epidemiology interventions must satisfy the unimpeachable goal of *primum on nocere* and the organizational mission of zero preventable harm. When applicable, employ abstracts, presentations, and published manuscripts from your home institution to drive ongoing institutional change (Authority), and present your lessons learned with enthusiasm and authenticity at the podium. Last, be clear on the tangible benefits of the proposed initiatives (Scarcity), these could include improvements in reported safety scores, benchmarking, and CMS reimbursements. When change implementation in healthcare epidemiology and antimicrobial stewardship is thoughtfully conceived and deliberately deployed, chances of success are optimized, but not guaranteed. The worst that can happen is that you “fail” to achieve the intended outcome; however, fear of failure should not be deterrent to improved patient safety.
